# Risk of Anaemia in Population of Healthy Young People Inhabiting a Region in Central Europe

**DOI:** 10.1155/2013/646429

**Published:** 2013-07-21

**Authors:** Małgorzata Szczuko, Izabela Gutowska, Teresa Seidler, Mariusz Mierzwa, Ewa Stachowska, Dariusz Chlubek

**Affiliations:** ^1^Department of Biochemistry and Human Nutrition, Pomeranian Medical University, Szczecin, Poland; ^2^Department of Human Nutrition, West Pomeranian University of Technology, Szczecin, Poland; ^3^Department of Biochemistry and Medical Chemistry, Pomeranian Medical University, Szczecin, Poland

## Abstract

It is quite unbelievable but significant percentage of young healthy women is at risk of anaemia despite proper nutritional state. In this study we decided to determine the lack or excess of which nutrients in a diet can lead to any effects. The major cause of anaemia is not, as in many studies on nutritional status, the deficiency in iron in a diet. Iron intake in women with anaemia exceeded AI (Adequate Intake) level. 120 people took part in the study. Average HGB concentration in female group amounted to 12.45 g/dL and in male group to 14.35 g/dL. Anaemia was determined in 29% women and 4.2% men. In group of women with anaemia a statistically higher intake of SFA, cholesterol, and sucrose and lower intake of pyridoxine, folacin, niacin, and vitamin E, Mn, Cu, Zn, Fe, and Mg were determined. In a group of men with low haemoglobin concentration a statistically higher intake of sucrose but lower intake of fat, especially SFA and MUFA, vitamin C and zinc were observed. Therefore, together with anaemia in the group of women there are coexisting deficiencies in other nutrients, as compared to the group of men. Prevention in both groups should include various supplements.

## 1. Introduction

Present-day diseases of affluence are often the effect of improper nutrition. It is considered that the fundamental cause of diet-related diseases is improperly balanced diet and wrong eating habits. Many experts think that young people cope well with excessive or deficient intake of nutrients. The aim of our study was to determine whether nutritional mistakes made in group of young educated people can be the cause of disorders and lead to diseases in their future life and the life of their children. It is especially important because whole Europe, as well as USA, deal more often with the issue of excessive intake of nutrients and increased percentage of obese people among the youth than with diseases connected to malnutrition [1, 2]. With respect to this, particularly dangerous is the deficiency in folacin in women's diets, particularly for women planning pregnancy, because it can lead to underdeveloped placenta and, in consequence, to miscarriage or neural tube defects in neonates [3]. In Poland, neural tube defects occur in 2-3 cases per 1000 births [4]. Another result of folacin deficiency is susceptibility of cells to neoplastic transformation, especially with respect to large intestine [5], increased risk of psychical disorders of depressive character [6], and occurrence of megaloblastic anaemia, characterized with lowered number of red cells simultaneously increased in size. We determined which nutrients are most responsible for changes in haemoglobin concentration in blood, considering sex and current eating trends among young people. The study involved the students of Food Technology and Human Nutrition, whose knowledge on proper diet is higher than in populations of students of other specializations. 

## 2. Material and Methods

### 2.1. Examined Group

The examination was carried out on students of Food Technology and Human Nutrition, volunteers, at the age of 22–24. Examined group comprised of 120 people, 96 women and 24 men. The study was approved by Bioethics Commission at the Pomeranian Medical University in Szczecin (N-001/12/06).

### 2.2. Interpretation of Haemoglobin and Hematocrit Tests Results

Reference ranges for HGB concentration (according to WHO definition) were considered as 12–16 mg/dL for women and 13–18 mg/dL for men. In examined group there were no people with anaemia.

Taking into consideration the results of complete blood count, the students were divided into three groups on the basis of haemoglobin concentration in blood, taking into account their sex. Different ranges of haemoglobin concentration were determined in female and male groups.

In the female group the ranges were as follows:

- I group with haemoglobin concentration <12 g/dL;
- II group with haemoglobin concentration 12-13 g/dL;
- III group with haemoglobin concentration >13 g/dL.

Among the men the following groups were distinguished:

- I group with haemoglobin concentration <14 g/dL;
- II group with haemoglobin concentration 14-15 g/dL;
- III group with haemoglobin concentration >15 g/dL.

In each of the previous groups of examined people, a nutritional assessment was made on the basis of nutrients content in 7-day food diary.

### 2.3. Blood Biochemical Tests

Blood was collected after fasting into a tube containing ethylenediaminetetraacetic acid (EDTA) as anticoagulant in form of film sprayed onto the walls of a tube for quantitative and qualitative analyses of blood morphotic elements. 

### 2.4. Computer and Statistical Analysis of Nutrition

In students' diets the assessment included the contents of energy, selected nutrients (protein, total fat, SFA, MUFA, and PUFA, and carbohydrates, including sucrose, vitamins A, D, E, C, B_1_, B_2_, B_6_, and B_12_, niacin, folacin, and mineral compounds: sodium, potassium, calcium, phosphorus, magnesium, iron, zinc, copper, manganese, and fibre, and cholesterol), and was performed using computer software “*Dietician 2*” and “*Food composition tables*” [7]. The calculation included correction indexes relating to losses occurring during storage and culinary treatment of food products.

To calculate significance of differences between the average values Tuckey's range test was used at the 0.05 level of significance (Statistica v 8.0). 

## 3. Results

### 3.1. Interview and General Examinations

Examined students formed a random group where average BMI (body mass index) for women was 21.2 and for men 23.4. Taking into consideration a normal BMI in young people ranging from 18.5 to 24.9 kg/m^2^ [8], in female group 81.2% women had proper body weight, and the same percentage (9.4%) had BMI <18.5 and BMI >24.9. In male group 66% population had proper body weight, 8% were men with BMI <18.5 and 25 with BMI >24.9. Average values of WHR (waist-hip ratio), which in women should reach ≤0.8 and in men ≤0.95, were 0.79 and 0.93, respectively.

### 3.2. Daily Food Rations (DFR) Composition with respect to Blood Morphology in Plasma (CBC)

**Figure 1:**
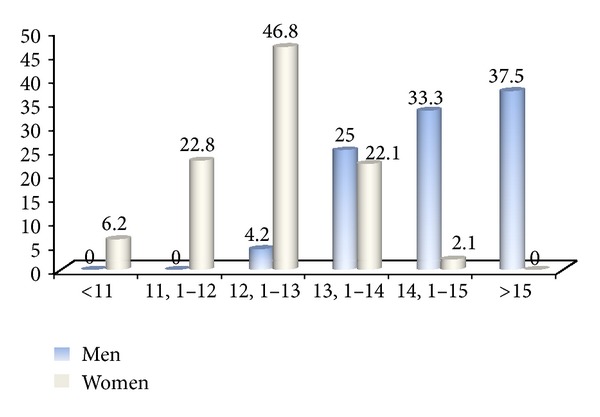
Percentage (%) of people examined in analysed ranges of haemoglobin concentration (g/dL).

Average values of selected blood morphology parameters in female and male groups were within reference ranges (Table 1). Average haemoglobin concentration (HGB), red blood count (RBC), packed cell volume (HCT), and mean cell haemoglobin (MCH) in women amounted to 12.45 g/dL, 4.3 M/ul, 37.9%, and 29.21 pico gram, respectively, whilst in men to 14.34 g/dL, 4.87 M/ul, 43.59% and 29.71 pico gram, respectively (Table 1). Statistical analysis of obtained results revealed that HGB and HCT in blood of men were significantly higher. It was determined that 29% women and only 4.2% men, according to WHO definition, had average HBG showing on anaemia (Figure 1). The highest percentage of women (46.8) had normal HGB concentration within the range from 12.1 g/dL to 13 g/dL, whereas in male group the distribution of HGB concentration levels within the three ranges (from 13.1 to 15.0 g/dL) was more even (Figure 1). 

### 3.3. Composition of Daily Food Rations (DFR) with respect to Haemoglobin Concentration in Blood

In the first group of women with the lowest haemoglobin concentration (HGB < 12 g/dL) a significantly higher intake of SFA (on average by 2.45 g/day), cholesterol (on average by 33.8 mg/day), and sucrose (on average by 13.2 g/day) was observed, as compared to the intake in the third group characterized by the highest level of haemoglobin (HGB > 13 g/dL) (Table 2). Moreover women with the highest level of haemoglobin in blood consumed significantly higher amounts of magnesium (on average by 23.5 and 24 mg/day), iron (on average by 1.16 mg/day in both groups), copper (on average by 0.25 and 0.23 mg), vitamin E (on average by 1 and 0.85 *μ*g/day), niacin (on average by 4.9 and 3.62 mg/day), and folacin (on average by 40.1 and 40.5 *μ*g/day) than women from other two groups and more zinc (on average by 0.86 mg/day), manganese (on average by 0.45 mg/day), and pyridoxine (on average by 0.2 mg/day) than women from the first group, and vitamin C (on average by 26.64 mg/day) than women from the second group. The remaining analysed nutrients (energy, protein, MUFA, PUFA, carbohydrates, lactose, dietary fibre, sodium, potassium, calcium, phosphorus, vitamins A and D, thiamine, riboflavin, and cobalamin) were consumed on similar levels in all respective groups of women (Table 2). In male population there were no such significant differences in DFR composition as in female population (Table 3). First group with the lowest haemoglobin level (HGB < 14 g/dL) was the most uniform group with respect to consumption of all of the analysed nutrients, as compared to other groups, and the analysed consumption was the lowest in this group. In the group with intermediate HGB concentration (14.1–15.0 g/dL) a significantly higher intake of fat (on average by 17.9 g/day), SFA (on average by 7.1 g/day) and MUFA (on average by 7.7 g/day), sodium (on average by 286 mg/day), zinc (on average by 1.7 mg/day), manganese (on average by 1.38 mg/day), and vitamin C (on average by 13.45 mg/day) was observed, as compared to the first group, and higher intake of sucrose was noted (on average by 17.5 g/day), as compared to the third group. In the latter group also a significantly higher intake of cobalamin (on average by 2.64–2.66 *μ*g/day) was observed in comparison to other groups (Table 3). The calculations of Tuckey's test parameters showed that HGB levels in women's blood were not related to energy structure of the diets (Table 2). In male group the percentage of energy from fat was higher in people with higher HGB level, and the percentage of energy from protein was higher in group with the highest HGB concentration (Table 3). 

## 4. Discussion

The analyses of HGB level in blood of examined students showed that in majority of people, it was normal. Considering the results with respect to sexes, it should be noted that in female group the percentage of women with normal HGB was lower by 24.8% than in men. But taking into consideration HGB values below 14 mg/dL in male group the difference would be compensated. Haemoglobin level in blood is the parameter widely used in medical diagnostics, in examination of general health state and nutritional status of individuals and whole populations, and also in context of living conditions [9, 10]. It is probably not such a huge problem as in India, where anaemia was observed in 54% population of students, and the cause was attributed to diet deficient in, respectively, folic acid, riboflavin, pyridoxine, vitamin C, and cobalamin. Interestingly, anaemia among young people in Turkey was caused by deficiency in iron and cobalamin but not folic acid [11]. The lowest levels of haemoglobin among healthy people are most commonly observed in pregnant women. It could be exemplified by women in Nepal, whose HGB concentration amounted, on average, to 10 mg/dL [12]. However, women taken part in this study werenot pregnant.

How did higher consumption of SFA, cholesterol, and sucrose affect lower HGB in women? It seems that the effect of these diet compounds is indirect, because these are the nutrients which in larger amounts are found in food rations of people with lower socioeconomic status. The sources of these nutrients are cheaper, less quality products, and thus of lower nutrition value. Murakami and co-authors [13], when assessing the effect of diet cost among almost 4 thousands Japanese female students, observed that higher cost of energy intake was connected to higher intake of fruits, sweetened drinks, oils, fish, crustaceans, and meat, the consequence of which was higher intake of fats (especially SFA), cholesterol, sodium, and animal protein. Higher consumption of mineral compounds in this study (including iron, magnesium, zinc, copper, and manganese), and also vitamin E, niacin, B6, folacin, and vitamin C, influenced the increase of HGB in blood. The effect of vitamin E, due to its antioxidative properties, increases the resistance of red blood cells to haemolysis and thus prevents the damage of capillary vessels walls [14]. Niacin, although it does not take part in erythropoiesis, as NAD+ and its phosphate (NADP+), but protects cell walls against oxidative damage. It reduces the oxidation of glutathione, which reduced form is engaged in regeneration of vitamin E [15]. Pyridoxine, as a coenzyme of delta-aminolevulinic acid synthetase, takes part in haemoglobin synthesis. Other studies also show that it inhibits superoxide radicals and prevents lipids peroxidation in erythrocytes [16]. Folacin, similarly as pyridoxine, cobalamin, and iron, participates in erythropoiesis [17]. Their main role is to deliver methylene group for synthesis of necessary thymidylate. The effect of vitamin C is unquestionable, since it facilitates iron assimilation and participates in haemoglobin synthesis and folic acid and copper conversion to active forms [18, 19].

In group of men some relations were similar as in female group. Sucrose again was consumed by people/subjects with the lowest HGB, what suggests that in both sexes the calorific value of the diet was complemented by sugar/sucrose and not by more valuable carbohydrate products, for example, wholemeal grains.

Rations of men with higher HGB level were richer in quality with respect to fat (SFA, MUFA), cholesterol, sodium, zinc, B12, and vitamin C, which probably had a similar role as in case of women in regulation of HGB concentration. A significant difference, however, was made by cobalamin, which, participating in conversion of propionic acid to succinyl-CoA, necessary in HGB synthesis and proper erythrocyte development, affected higher HBG concentration. In addition, two other factors may have an impact on anaemia in this group of women. First, heavy menstrual bleeding could lead to significant loss of iron from the body [20]. Secondly, the use of contraception, confirmed by 39% of women participating in the study, may reduce the absorption of vitamins, including vitamins involved in erythropoiesis. Additionally, some authors indicated that viral infections, especially of the Parvoviridae family [21] and autoimmunology diseases [22], also are the factors affecting the occurrence of anaemia. However, these factors could not be taken into account in our study.

## 5. Conclusions

1. In group of young people from Poland to lower anaemia the following intervention should be made:
  - first of all, to replenish the deficiency in folacin in a diet,
  - next, to eliminate sucrose as the source of energy and replace it with wholemeal grain products containing mineral compounds and pyridoxine,
  - then, to complement the diet with vitamin C,
  - and to take into consideration higher supply of mineral compounds (Mg, Fe, Zn, Cu, and Mn), vitamin E, and niacin, especially among women.
2. Iron consumption among young women at the level of 8.91 mg/day (with AI (Adequate Intake) level being 8 mg/day), with simultaneous deficiency in other nutrients participating in erythropoiesis, is not sufficient in anaemia prevention in this group.

## Figures and Tables

**Table 1:** Average values of selected blood morphology parameters.

Parameters	Women		Men	
	$\overset{-}{x}$	SD	$\overset{-}{x}$	SD
HGB (g/dL)	12.45*	0.86	14.34*	1.37
RBC (M/uL)	4.30	0.33	4.87	0.49
HCT (%)	37.9*	2.45	43.59*	3.87
MCH (pg)	29.21	2.23	29.71	1.00

*Statistically significant difference with *P* < 0.05; %: percentage of people with proper levels of lipid profile parameters; $\overset{-}{x}$: average content in a group.

**Table 2:** Women's DFR composition with respect to haemoglobin concentration in blood.

Parameter	Haemoglobin concentration ranges (g/dL)					
	HGB < 12 g/dL		12.1–13.0 g/dL		HGB > 13.0 g/dL	
	$\overset{-}{x}$	SD	$\overset{-}{x}$	SD	$\overset{-}{x}$	SD
Energy (kcal)	1763^a^	708.5	1729^a^	292.7	1730^a^	325.4
Protein (g)	60.87^a^	22.55	61.94^a^	12.85	61.92^a^	10.46
Fat (g)	68.45^a^	27.52	69.02^a^	15.84	65.06^a^	16.59
SFA (g)	25.56^b^	11.37	25.20^ab^	6.37	23.11^a^	8.01
MUFA (g)	27.42^a^	11.01	28.09^a^	6.86	25.58^a^	7.10
PUFA (g)	10.33^a^	3.45	10.42^a^	3.18	11.24^a^	5.06
Cholesterol (mg)	241.6^b^	102.07	242.3^b^	73.05	207.8^a^	71.91
Carbohydrates (g)	235.3^a^	95.31	226.6^a^	42.25	228.3^a^	50.03
Sucrose (g)	68.01^b^	34.30	55.43^a^	21.22	54.84^a^	17.08
Lactose (g)	8.54^a^	4.70	7.93^a^	3.98	8.65^a^	3.83
Dietary fibre (g)	15.37^a^	5.34	15.74^a^	3.80	15.97^a^	3.69
Na (mg)	1684^a^	567.17	1804^a^	600.59	1824^a^	323.58
K (mg)	2368^a^	990.06	2372^a^	454.76	2462^a^	727.50
Ca (mg)	596.2^a^	247.38	549.2^a^	178.75	568.5^a^	214.10
P (mg)	1003^a^	367.57	1019^a^	214.27	1039^a^	174.92
Mg (mg)	231.6^a^	97.54	232.1^a^	43.96	255.6^b^	60.35
Fe (mg)	8.91^a^	3.27	8.91^a^	1.65	10.07^b^	4.50
Zn (mg)	7.92^a^	2.70	8.13^ab^	1.66	8.78^b^	4.39
Cu (mg)	0.96^a^	0.35	0.94^a^	0.18	1.19^b^	0.58
Mn (mg)	3.70^a^	1.25	3.81^ab^	1.01	4.15^b^	1.77
Vitamin A (*µ*g)	990.3^a^	1586	863.9^a^	935.44	1012^a^	1465
Vitamin D (*µ*g)	2.83^a^	1.66	2.73^a^	1.96	2.55^a^	1.80
Vitamin E (mg)	7.36^a^	2.50	7.52^a^	2.19	8.37^b^	4.04
Vitamin B1 (mg)	0.84^a^	0.33	0.93^a^	0.31	0.87^a^	0.13
Vitamin B2 (mg)	1.24^a^	0.43	1.29^a^	0.43	1.32^a^	0.16
Niacin (mg)	11.98^a^	5.56	13.26^a^	4.40	16.88^b^	12.01
Vitamin B6 (mg)	1.35^a^	0.57	1.53^b^	0.49	1.55^b^	0.85
Folacin (*µ*g)	145.5^a^	68.82	145.1^a^	41.89	185.6^b^	122.9
Vitamin B12 (*µ*g)	3.41^a^	3.70	3.45^a^	2.84	4.32^a^	7.95
Vitamin C (mg)	52.66^ab^	104.18	39.02^a^	19.43	65.66^b^	103.7
% Energy from protein	13.6^a^	2.96	14.1^a^	3.05	14.1^a^	3.08
% Energy from fat	35.5^a^	6.39	36.5^a^	6.35	34.4^a^	6.11
% Energy from carbohydrates	50.9^a^	6.24	49.4^a^	8.37	51.5^a^	6.33

^a,b^Homogenous groups according to Tuckey's test.

**Table 3:** Men's DFR composition with respect to haemoglobin concentration in blood.

Parameter	Haemoglobin concentration ranges (g/dL) *n* = 24					
	HGB < 14 g/dL		14.1–15.0 g/dL		HGB > 15.0 g/dL	
	$\overset{-}{x}$	SD	$\overset{-}{x}$	SD	$\overset{-}{x}$	SD
Energy (kcal)	2159^a^	724.6	2364^a^	496.06	2308^a^	707.2
Protein (g)	80.32^a^	27.53	79.88^a^	15.24	80.09^a^	15.73
Fat (g)	83.06^a^	34.30	101.0^b^	19.32	96.76^ab^	36.23
SFA (g)	29.68^a^	15.22	36.77^b^	8.87	36.13^ab^	16.23
MUFA (g)	33.01^a^	11.85	40.73^b^	7.20	39.54^ab^	15.14
PUFA (g)	14.10^a^	4.92	15.67^a^	5.05	13.65^a^	2.79
Cholesterol (mg)	280.8^a^	109.5	290.9^ab^	112.59	359.4^b^	185.8
Carbohydrates (g)	289.1^a^	90.20	305.2^a^	77.75	287.4^a^	63.64
Sucrose (g)	61.23^ab^	23.29	75.23^b^	21.37	57.72^a^	14.16
Lactose (g)	12.79^a^	8.54	10.02^a^	7.35	11.68^a^	7.47
Dietary fibre (g)	18.91^a^	9.87	22.13^a^	9.53	18.26^a^	4.16
Na (mg)	2122^a^	911.7	2448^b^	577.4	2568^b^	849.6
K (mg)	3022^a^	1270	3156^a^	1077	2724^a^	621.3
Ca (mg)	797.1^a^	565.3	698.6^a^	150.8	722.1^a^	319.8
P (mg)	1330^a^	603.5	1361^a^	310.7	1323^a^	257.9
Mg (mg)	291.8^a^	147.27	320.2^a^	119.5	276.9^a^	49.10
Fe (mg)	11.14^a^	4.11	12.73^a^	2.53	11.35^a^	2.54
Zn (mg)	10.05^a^	2.82	11.75^b^	2.83	10.76^ab^	2.17
Cu (mg)	1.19^a^	0.64	1.23^a^	0.52	1.07^a^	0.18
Mn (mg)	4.53^a^	1.78	5.91^b^	2.30	4.32^a^	1.60
Vitamin A (*µ*g)	832.4^a^	971.54	1015^a^	605.26	967.4^a^	990.6
Vitamin D (*µ*g)	2.87^a^	0.97	3.71^a^	2.34	3.95^a^	2.36
Vitamin E (mg)	9.44^a^	5.14	8.97^a^	4.40	9.05^a^	1.63
Vitamin B1 (mg)	1.20^a^	0.45	1.43^a^	0.46	1.27^a^	0.34
Vitamin B2 (mg)	1.63^a^	0.74	1.61^a^	0.31	1.74^a^	0.57
Niacin (mg)	18.30^a^	5.36	16.76^a^	4.20	15.62^a^	3.39
Vitamin B6 (mg)	2.08^a^	0.68	1.72^a^	0.59	1.80^a^	0.52
Folacin (*µ*g)	171.5^a^	40.48	183.4^a^	50.11	184.9^a^	64.90
Vitamin a B12 (*µ*g)	3.24^a^	1.63	3.26^a^	0.77	5.90^b^	2.44
Vitamin C (mg)	42.92^a^	29.79	56.37^b^	24.44	54.16^ab^	38.64
% Energy from protein	14.7^b^	3.26	13.3^a^	3.05	13.7^ab^	3.78
% Energy from fat	35.0^a^	8.39	39.0^b^	6.35	38.3^b^	7.11
% Energy from carbohydrates	50.3^a^	8.24	47.7^a^	8.37	48.0^a^	8.33

^a,b^Homogenous groups according to Tuckey's test.
